# Ketogenic Dietary Therapies and β-Hydroxybutyrate as Modulators of Mitochondrial Function: From Molecular Mechanisms to Clinical Translation

**DOI:** 10.3390/nu18193256

**Published:** 2026-10-02

**Authors:** Ji-Hoon Na, Young-Mock Lee

**Affiliations:** Division of Pediatric Neurology, Department of Pediatrics, Gangnam Severance Hospital, Yonsei University College of Medicine, Seoul 06273, Republic of Korea; jhnamd83@yuhs.ac

**Keywords:** diet, ketogenic, 3-hydroxybutyric acid, mitochondria, drug resistant epilepsy, mitochondrial diseases, lysine β-hydroxybutyrylation, histone deacetylase inhibition, ketone ester

## Abstract

Ketogenic dietary therapies (KDTs) have been used for a century in drug-resistant epilepsy, and β-hydroxybutyrate (BHB), the major ketone body, is recognized as a signaling metabolite. This review examines how KDTs and BHB modulate mitochondrial function and evaluates evidence supporting these mechanisms. BHB is oxidized through D-β-hydroxybutyrate dehydrogenase 1 and succinyl-CoA:3-oxoacid-CoA transferase and alters redox balance and oxidative phosphorylation. It also inhibits class I histone deacetylases, serves as the substrate for lysine β-hydroxybutyrylation, activates hydroxycarboxylic acid receptor 2, and blocks the NLR family pyrin domain-containing 3 inflammasome. These pathways influence redox defense, organelle quality, and inflammation. However, ketogenic feeding suppresses mitochondrial one-carbon metabolism and some signaling effects occur only at BHB concentrations exceeding those typically achieved through dietary ketosis. Clinical benefits are best established in drug-resistant epilepsy and appear genotype-dependent in primary mitochondrial disease, with potential benefit in mitochondrial DNA (mtDNA) variant carriers but possible harm in DNA polymerase gamma-related disease and mtDNA deletion myopathy. Outside neurology, human evidence includes a hemodynamic response to infused 3-hydroxybutyrate in heart failure, sustained glycemic improvement in non-randomized diabetes cohorts, and small trials in psychiatric and oncological populations. Most clinical studies have not directly measured mitochondrial outcomes. Proposed mitochondrial mechanisms of KDTs and BHB remain plausible but are not established explanations for clinical benefit. Genotype-stratified trials, ketone exposure assessment, and direct measurement of mitochondrial variables in patients are needed.

## 1. Introduction

Fasting has been used as a treatment for seizures since the Hippocratic era, but dietary therapy in its modern form dates to 1921, when Wilder proposed at the Mayo Clinic that the benefits of starvation could be reproduced if ketonemia was induced by other means and coined the term ketogenic diet (KD) [1]. A century later, ketogenic dietary therapies (KDTs), including the classic KD, modified Atkins diet (MAD), medium-chain triglyceride (MCT) diet, and low glycemic index treatment (LGIT), are delivered under international consensus protocols specifying patient selection, initiation, supplementation, and monitoring [2]. Controlled evidence began with the first randomized trial, in which 38% of children assigned to the KD group achieved more than 50% seizure reduction at 3 months compared with 6% of controls. The variants were subsequently compared directly in children with drug-resistant epilepsy and in a network meta-analysis [3,4,5]. Approximately 48% of treated individuals achieve at least a 50% reduction in seizure frequency or seizure freedom, yet the certainty of randomized evidence is rated as limited, and the mechanisms producing this benefit remain incompletely defined [6].

β-Hydroxybutyrate (BHB) is the quantitatively dominant ketone body, synthesized in hepatic mitochondria from acetyl-coenzyme A (acetyl-CoA) derived from fatty acid oxidation and exported for terminal oxidation in extrahepatic tissues [7]. Its biological status has changed substantially. BHB is an endogenous and selective inhibitor of class I histone deacetylases (HDACs), and its administration induces the oxidative stress resistance genes *Foxo3a* and metallothionein 2 [8]. It is also the substrate for lysine β-hydroxybutyrylation and a ligand for the surface receptors hydroxycarboxylic acid receptor 2 (HCAR2) and free fatty acid receptor 3 (FFAR3) [9]. Concentrations sufficient for these actions, in the range of 1–2 mmol/L, are reached during fasting, sustained exercise, and dietary therapy [8,10].

These actions converge at the mitochondria. Bioenergetic failure is recognized as a shared mechanism across neurological disorders, and the neuroprotective effects of ketogenic therapies have been attributed to improved mitochondrial metabolism [11]. Mitochondrial dysfunction has since become an explicit therapeutic target across organ systems, although human studies are scarce relative to the preclinical literature [12]. Primary mitochondrial diseases are the most common group of inherited metabolic disorders with a minimum birth prevalence of approximately 20 per 100,000 [13]. KDTs have been reported to perform better than the average dietary response in some patients with complex I mitochondrial disorders; however, systematic appraisal indicates that efficacy and safety data in mitochondrial disease remain sparse and genotype-dependent [2,14].

Ketosis and carbohydrate restriction can be dissociated in humans. Ingestion of an (R)-3-hydroxybutyrate ketone ester raises blood D-BHB from approximately 0.1 to 3.5 mmol/L within 10 min without carbohydrate restriction, making BHB exposure a titratable variable rather than an inevitable consequence of diet composition [10]. We previously reviewed KDTs in genetically confirmed drug-resistant epilepsy and argued that genotype should inform patient selection, while the signaling actions of BHB, the consequences of sustained ketosis, and the role of exogenous ketones warrant further investigation [15]. The present review examines these issues by considering how KDTs and BHB modulate mitochondrial substrate handling, redox state, epigenetic regulation, receptor signaling, quality control, and inflammation, and assessing which of these mechanisms have been corroborated in patients rather than solely in cells and animals. Discordant observations, such as the suppression of mitochondrial one-carbon metabolism during ketogenic feeding, are considered in addition to the supportive data [16]. We aimed to distinguish mechanisms established in humans from those inferred from preclinical models and identify the measurements required to close that distance.

## 2. Ketone Body Metabolism at the Mitochondrion

**Figure 1:**
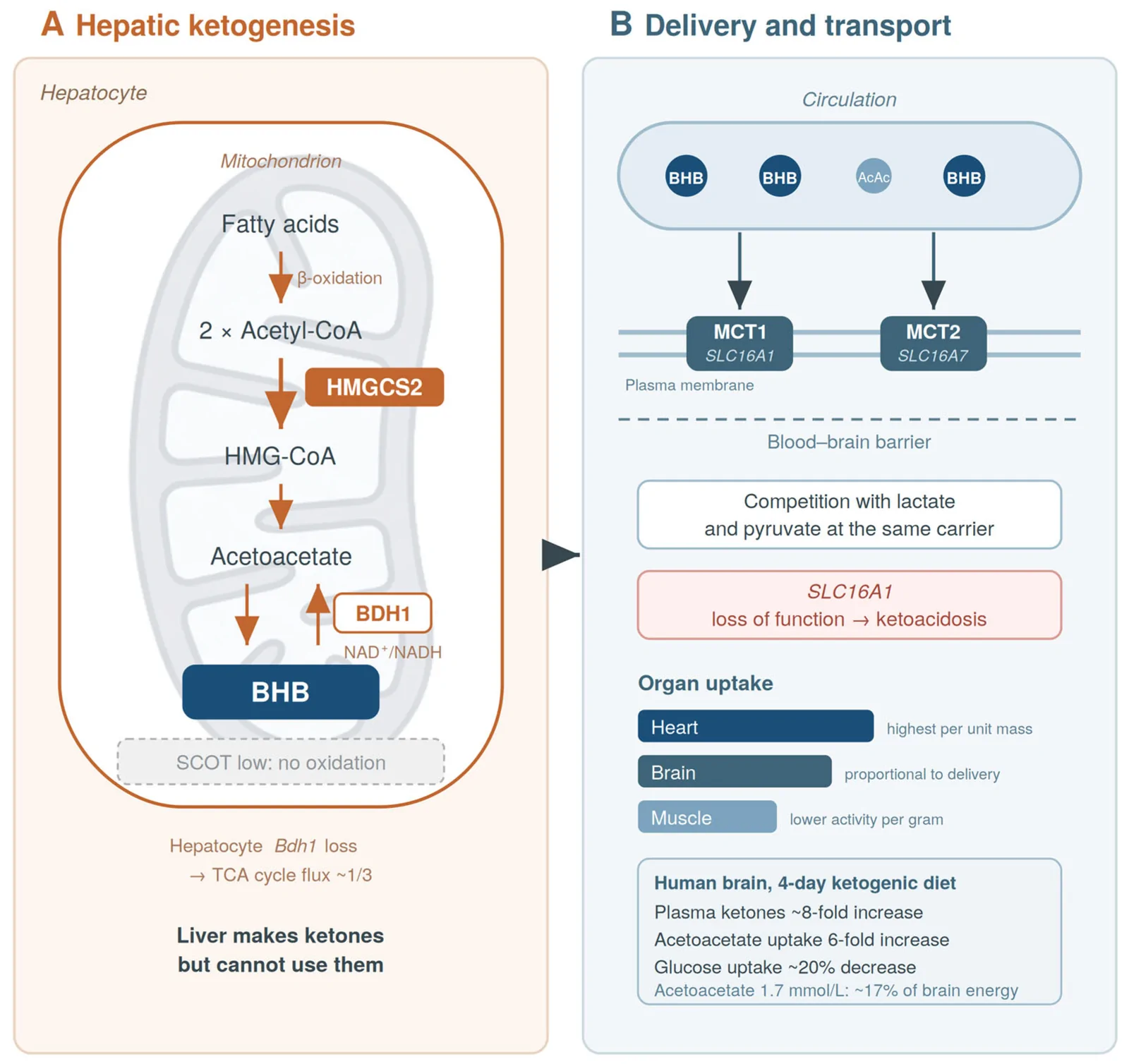
Hepatic ketogenesis and the delivery of β-hydroxybutyrate to extrahepatic tissues. Values shown are those reported in the studies cited in the main text. (**A**) Hepatic mitochondria condense fatty acid–derived acetyl-CoA through a pathway controlled by HMGCS2, and BDH1 then reduces acetoacetate to BHB in a near-equilibrium reaction coupled to the mitochondrial NAD^+^/NADH ratio. Hepatocytes express little SCOT and therefore export ketones rather than oxidize them; in hepatocyte-specific *Bdh1*-knockout mice, TCA cycle flux falls to about one-third of control. (**B**) MCT1 and MCT2 carry ketones across the plasma membrane and the blood–brain barrier in competition with lactate and pyruvate, and inactivating SLC16A1 variants cause ketoacidosis in patients. Bar lengths indicate the relative importance of each organ as a ketone consumer rather than a measured flux. Boxed values are from positron emission tomography in healthy adults after 4 days of a ketogenic diet. Gene names are italicized and protein names are in roman type. AcAc, acetoacetate; BDH1, D-β-hydroxybutyrate dehydrogenase 1; BHB, β-hydroxybutyrate; HMG-CoA, 3-hydroxy-3-methylglutaryl-CoA; HMGCS2, 3-hydroxy-3-methylglutaryl-CoA synthase 2; MCT1, monocarboxylate transporter 1; MCT2, monocarboxylate transporter 2; NAD, nicotinamide adenine dinucleotide; SCOT, succinyl-CoA:3-oxoacid-CoA transferase; TCA, tricarboxylic acid.

Ketone body metabolism involves a division of labor between the liver, which produces ketones but cannot oxidize them, and extrahepatic tissues, which use them as fuel (Figure 1) [7]. During fasting or carbohydrate restriction, hepatic mitochondria convert fatty acid-derived acetyl-CoA to ketone bodies through a three-step pathway; the rate-controlling enzyme in this pathway is 3-hydroxy-3-methylglutaryl-CoA synthase 2. Acetoacetate is then reduced to BHB, and both ketones enter the circulation [7,17]. BHB predominates in blood, whereas acetoacetate is more readily drawn into terminal oxidation, and the BHB-to-acetoacetate ratio reflects tissue redox state [18]. Once viewed only as starvation substrates, ketones are now recognized as metabolic and signaling mediators even when carbohydrates are available [7,9]. The enzymes and transporters involved in ketone production, delivery, and oxidation determine tissue BHB availability and utilization, whereas defects in individual steps can cause distinct diseases [7,17].

The interconversion of acetoacetate and BHB is a redox-coupled reaction that links ketone metabolism to the mitochondrial nicotinamide adenine dinucleotide (NAD^+^)/NADH ratio [18]. D-β-hydroxybutyrate dehydrogenase 1 (BDH1) catalyzes this near-equilibrium step; therefore, the acetoacetate-to-BHB ratio reflects the compartmental redox state, and the two ketones behave as a linked redox pair rather than independent substrates [17,18]. This coupling helped prompt the reappraisal of ketones as signals rather than as inert fuels [9]. In hepatocyte-specific *Bdh1*-knockout mice, the liver can no longer interconvert the two ketones, reducing tricarboxylic acid (TCA) cycle flux to approximately one-third of control levels and impairing gluconeogenesis, although whole-body equilibration is preserved by extrahepatic BDH1 [18]. BDH1 therefore influences hepatic redox state and oxidative flux, rather than merely producing fuel, consistent with the long-recognized dependence of ketone handling on tissue-specific enzyme expression [7,17].

Terminal oxidation in extrahepatic tissues depends on a dedicated activation step that the liver lacks [7]. Succinyl-CoA:3-oxoacid-CoA transferase (SCOT, encoded by *OXCT1*) activates acetoacetate for cleavage into two acetyl-CoA units that enter the TCA cycle, whereas hepatocytes express little SCOT and therefore export rather than consume ketones [7,17]. The physiological importance of this step is shown in *Oxct1*-deficient mice, which are born normally but die within two days of birth from ketoacidosis and hypoglycemia because ketones cannot enter the TCA cycle [19]. Their brain and skeletal muscles mount distinct compensatory increases in glucose and lactate oxidation; therefore, ketolytic capacity is tissue-specific rather than uniform [17,19]. In humans, SCOT deficiency presents early in life with severe ketoacidosis, lethargy, vomiting, and coma. These findings demonstrate that this activation step is essential for ketone utilization in humans [7].

The delivery of ketones into cells is governed by monocarboxylate transporters, and here, the evidence is derived directly from humans. Proton-linked transporters of the *SLC16* family, principally MCT1 (*SLC16A1*) and MCT2 (*SLC16A7*), carry BHB and acetoacetate across the plasma membrane and blood–brain barrier [7]. Because the same carriers also move lactate and pyruvate, ketone uptake is subject to substrate competition when glycolytic flux is high [10]. Biallelic and even heterozygous inactivating *SLC16A1* mutations cause recurrent and sometimes profound ketoacidosis with severity-tracking transport capacity, showing that MCT1-mediated import is required for acid–base balance and that impaired delivery alone can drive decompensation [20]. Transporter abundance is also inducible; therefore, uptake capacity increases with sustained exposure, a property relevant to adaptation to prolonged dietary therapy [17,21].

The importance of this uptake differs sharply among organs, with the heart and brain being the principal ketone consumers [17]. The myocardium oxidizes ketones in proportion to delivery and is the highest consumer per unit mass, while skeletal muscle contributes through its large mass despite its lower per-gram activity [7,17]. In humans, acute BHB elevation shifts the muscle fuel selection away from glycolysis, confirming that these organs use ketones at physiological concentrations [10]. In healthy adults, dual-tracer positron emission tomography of the brain showed that 4 days of a KD raised plasma ketones about eight-fold, increased the cerebral metabolic rate of acetoacetate six-fold, and lowered that of glucose by about 20%; uptake rose with plasma concentration, and a blood acetoacetate concentration of 1.7 mmol/L was estimated to supply about 17% of whole-brain energy needs [21]. Concentrations within this range are achieved with dietary therapy and exogenous ketones [10].

## 3. Molecular Mechanisms Linking BHB to Mitochondrial Function

### 3.1. Bioenergetics and the Mitochondrial Redox Couple

**Figure 2:**
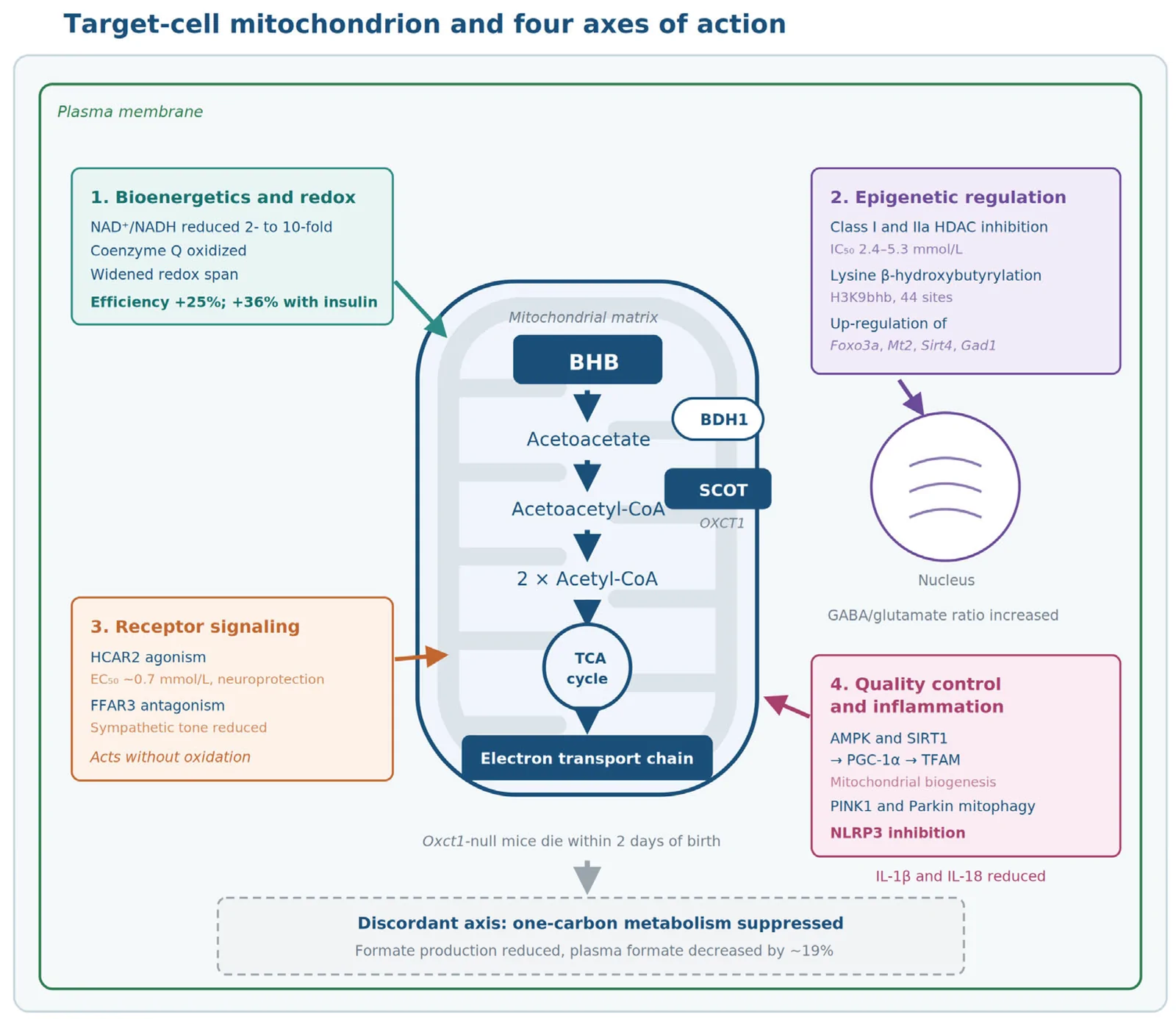
Four axes through which β-hydroxybutyrate acts on the target-cell mitochondrion. Values shown are those reported in the studies cited in the main text, and the experimental system is indicated because most mechanistic data derive from animal or cell models rather than from patients. BHB is oxidized through BDH1 and SCOT into the TCA cycle, a step shown to be obligatory by the neonatal death of *Oxct1*-null mice. Efficiency values in axis 1 are from the isolated working rat heart. In axis 2, the median inhibitory concentrations for the class I enzymes HDAC1 and HDAC3 and for the class IIa enzyme HDAC4 are from purified-enzyme assays, lysine β-hydroxybutyrylation was mapped in human cells and mouse liver, and the transcriptional changes shown were observed in mice. In axis 3, the half-maximal effective concentration for HCAR2 lies within the physiological range, and this axis operates at the plasma membrane without requiring ketone oxidation. Axis 4 shows engagement of AMPK and SIRT1, PINK1- and Parkin-dependent mitophagy, and inhibition of the NLRP3 inflammasome. The grey dashed axis marks a discordant effect observed in HepG2 cells and in mice fed a medium-chain-triglyceride ketogenic diet. Gene names are italicized and protein names are in roman type. AMPK, AMP-activated protein kinase; BDH1, D-β-hydroxybutyrate dehydrogenase 1; BHB, β-hydroxybutyrate; EC50, half-maximal effective concentration; FFAR3, free fatty acid receptor 3; GABA, γ-aminobutyric acid; HCAR2, hydroxycarboxylic acid receptor 2; HDAC, histone deacetylase; IC50, median inhibitory concentration; IL, interleukin; NLRP3, NLR family pyrin domain containing 3; PGC-1α, peroxisome proliferator-activated receptor gamma coactivator 1-alpha; PINK1, PTEN-induced kinase 1; SCOT, succinyl-CoA:3-oxoacid-CoA transferase; SIRT1, sirtuin 1; TCA, tricarboxylic acid; TFAM, mitochondrial transcription factor A.

The most direct way in which BHB acts on the mitochondria is by improving the thermodynamic efficiency of oxidative phosphorylation (Figure 2). In isolated working rat hearts, adding a physiological ratio of ketone bodies to a glucose-containing buffer increased the hydraulic efficiency by 25%, and combining ketones with insulin increased it by 36% [22]. These gains were attributed to a two- to tenfold reduction in the mitochondrial NAD^+^/NADH couple and oxidation of the coenzyme Q couple, which widened the redox span across the electron transport chain and increased the free energy available from adenosine triphosphate (ATP) hydrolysis [7,22]. Since ketone oxidation keeps ubiquinone more oxidized than fatty acid oxidation does, it yields more energy per molecule of oxygen consumed and may compensate for defects in mitochondrial energy transduction [11,22]. This principle has since been examined in cultured neurons rather than perfused organs. In primary mouse cortical neurons maintained at physiological oxygen tension, 5 mmol/L BHB sustained a higher proportion of oxygen consumption devoted to ATP synthesis and supported stronger and more sustained maximal uncoupled respiration than glucose, an effect most evident under glutamate-mediated excitotoxic stress [23].

Beyond increasing efficiency, ketone metabolism reshapes flux through the TCA cycle. Ketogenic states restrict glycolysis and increase fatty acid oxidation. The resulting acetyl-CoA is oxidized in the cycle and sustains the flux needed for continued respiration, whereas replenishment of the cycle intermediates depends on anaplerotic substrates that ketone bodies do not supply [7,11]. This is important when glucose handling is impaired because ketones enter oxidation downstream of glycolysis and the pyruvate dehydrogenase step, bypassing reactions that fail in several metabolic epilepsies [7,11]. In mouse cortical neurons, the respiration supported by BHB is directed towards ATP synthesis to a greater extent than that supported by glucose, and the difference is largest when glutamate raises energetic demand [23]. Substrate choice therefore bears on the capacity to buffer excitatory stress, which is relevant where oxidative capacity is already reduced, as it is in epileptic tissue [11]. The magnitude of these effects depends on the amount of ketones reaching the tissue, which is determined by transporter capacity and competition with lactate and pyruvate [7,17].

### 3.2. Defense Against Oxidative Stress

A second mechanism follows from the redox effects described above; by altering the electron flow, BHB lowers the production of reactive oxygen species (ROS) and simultaneously strengthens the cell’s antioxidant machinery. Uncoupling proteins induced during ketogenic feeding dissipate a portion of the proton gradient, which reduces electron leakage and superoxide formation while preserving respiration [11]. Mitochondrial ROS largely arise from a highly reduced electron transport chain; therefore, the more oxidized quinone pool favored by ketones lowers the driving force for ROS formation [22]. Simultaneously, ketone metabolism activates the SIRT3–FOXO3a axis and its downstream targets, manganese superoxide dismutase and catalase, coordinating a transcriptional program that increases oxidative buffering [24,25,26]. These effects have been characterized in rodent tissues; however, their magnitude in human mitochondria remains to be established [12].

The clearest evidence for this defense comes from a study showing that BHB induces oxidative stress resistance genes through an epigenetic rather than a purely metabolic route. In mice, exogenous BHB, fasting, or caloric restriction increased the transcription of *Foxo3a* and metallothionein 2, and chromatin immunoprecipitation showed increased histone acetylation at the promoters of both genes [8]. Metallothioneins scavenge ROS directly, whereas FOXO3a is a transcription factor that drives the expression of antioxidant enzymes; therefore, their induction provides a plausible route from ketosis to oxidative defense [25]. Selective depletion of HDAC1 and HDAC2 reproduced this induction, and mice pretreated with BHB were substantially protected against the oxidative toxin paraquat, with reduced protein carbonylation in the kidney tissue [8]. This links the antioxidant action of BHB to the inhibition of specific deacetylases rather than to its role as a substrate, identifying the FOXO3a network as an effector [24,26]. By protecting the mitochondrial permeability transition pore and limiting caspase activation, the same pathway also restrains the apoptotic consequences of oxidative injury, allowing BHB to protect cells even when its contribution as a fuel is modest [11,24,26].

### 3.3. Epigenetic Regulation: HDAC Inhibition and Lysine β-Hydroxybutyrylation

The observation that BHB acts through deacetylases points to a broader epigenetic role, which is now among the best-characterized non-metabolic functions of this molecule. BHB is an endogenous and selective inhibitor of zinc-dependent HDACs: in vitro it inhibits the class I enzymes HDAC1 and HDAC3 with median inhibitory concentrations of 5.3 and 2.4 mmol/L and the class IIa enzyme HDAC4 at 4.5 mmol/L, concentrations attainable in human blood during fasting or sustained exercise [8,9]. Inhibition begins at 1–2 mmol/L and does not extend to the class IIb deacetylase HDAC6, which required 48.5 mmol/L, indicating a selective rather than general effect [8]. Since class I HDACs normally suppress transcription, their inhibition by BHB shifts a defined set of genes toward activation, with competitive occupancy of the catalytic site likely underlying this effect [9]. Independent studies in brain tissue have confirmed that dietary BHB inhibits HDAC1 and HDAC2 and increases histone acetylation in vivo, extending the findings from purified enzymes to intact organs [27].

A distinct epigenetic action of BHB is that it serves as a substrate for protein modification. Lysine β-hydroxybutyrylation (Kbhb) was identified as a histone mark induced in proportion to circulating BHB, with 44 sites detected across human cells and mouse liver, a number comparable to that of histone acetylation [28]. This suggests that the modification is a broad regulatory system, rather than an incidental adduct [9]. Chromatin studies have shown that H3K9bhb is enriched at active gene promoters and that its increase during starvation is associated with the upregulation of starvation-responsive metabolic genes, establishing Kbhb as a marker that couples metabolism to gene expression [25,28]. The modification extends beyond histones because BHB-driven Kbhb of the tumor suppressor p53 and of metabolic enzymes alters their activity. In cancer models, diet-induced Kbhb of aldolase B suppresses mammalian target of rapamycin signaling and cell proliferation [25,29]. Therefore, Kbhb acts alongside HDAC inhibition rather than through it [9,29].

The epigenetic actions of BHB may contribute to a specific neurochemical outcome relevant to its anti-seizure effects. In a pentylenetetrazole model, ketogenic-diet-derived BHB, but not other ketone bodies, inhibited HDAC1 and HDAC2, increased H3K27 acetylation, and transcriptionally upregulated sirtuin 4 and glutamate decarboxylase 1 [27]. This transcriptional route is consistent with the downstream effects of class I HDAC inhibition described above, now acting on genes that govern inhibitory neurotransmission [28]. The induced sirtuin 4 protein inactivated glutamate dehydrogenase, preserving glutamate for conversion to γ-aminobutyric acid (GABA), whereas glutamate decarboxylase 1 upregulation increased the brain GABA/glutamate ratio and reduced neuronal excitation [27]. Since the antiseizure benefit depends on an elevated GABA/glutamate ratio, this pathway provides a potential link between the epigenetic action of BHB and a measurable functional endpoint, although these findings do not establish that BHB itself, rather than the diet as a whole, is responsible for the clinical effect [9,27].

### 3.4. Surface Receptor Signaling Through HCAR2 and FFAR3

BHB also acts without entering the cell by binding to G-protein-coupled receptors on the plasma membrane, which distinguishes it from conventional fuels that must be oxidized to have an effect. Two receptors are established targets: HCAR2 (also called GPR109A) and FFAR3 (also called GPR41), both of which couple with inhibitory G proteins and modulate metabolism and inflammation [9,30]. HCAR2 was first identified as a nicotinic acid receptor and later shown to be activated by BHB with a half-maximal effective concentration of about 0.7 mmol/L, a value within the physiological range of ketosis [9]. Functional consequences have been demonstrated directly in the brain, where the neuroprotective effects of BHB and a KD against ischemic stroke are lost in *Hcar2*-deficient mice, establishing the receptor as a necessary mediator rather than an incidental target [31]. Activation of HCAR2 in infiltrating monocytes and macrophages drives them toward a neuroprotective phenotype dependent on prostaglandin production, providing a defined cellular route from circulating metabolites to tissue protection [30,31].

For the second receptor, BHB acts as an antagonist rather than an agonist. BHB antagonizes FFAR3 in sympathetic neurons, suppressing propionate-induced activation of extracellular signal-regulated kinase signaling and the associated increase in heart rate, an effect absent in *Ffar3*-deficient mice [32]. This antagonism links ketosis to a reduction in sympathetic tone during fasting, which aligns energy expenditure with nutrient availability [9,32]. The direction of BHB action at FFAR3 remains controversial because other studies using rat neurons have reported agonism and inhibition of *N*-type calcium channels, a discrepancy that may reflect species differences or partial agonism [30]. Regardless of the direction, receptor-level signaling operates at concentrations achievable through dietary therapy and adds a membrane-delimited layer to the actions of BHB, which is independent of its oxidation [9,31]. These receptors are attractive sites for reproducing the effects of ketosis pharmacologically without dietary restrictions [30].

### 3.5. Mitochondrial Quality Control: Biogenesis, Dynamics, and Mitophagy

The mechanisms described thus far change the behavior of existing mitochondria, but ketone metabolism also alters the size, number, and turnover of the mitochondrial population. Mitochondrial biogenesis is governed by the peroxisome proliferator-activated receptor gamma coactivator 1-alpha, which recruits nuclear respiratory factors and mitochondrial transcription factor A to coordinate the expression of nuclear and mitochondrial genes required to build new organelles [33]. The two principal upstream activators of this program are AMP-activated protein kinase (AMPK) and NAD^+^-dependent deacetylase SIRT1, both of which are involved in the energetic and redox shifts that accompany ketosis [12,33]. Because ketone oxidation lowers the NAD^+^/NADH ratio in some compartments while raising cytosolic NAD^+^ availability elsewhere, it is positioned to activate SIRT1 and, in turn, to deacetylate and activate peroxisome proliferator-activated receptor gamma coactivator 1-alpha [11]. KDs and their variants have been reported to enhance markers of biogenesis in the brain and other tissues, consistent with increased mitochondrial content rather than improved efficiency of individual organelles [11,26].

Quality control extends beyond building new mitochondria to the remodeling and removal of damaged mitochondria. Mitochondrial dynamics, defined by the balance between fission mediated by dynamin-related protein 1 and fusion mediated by mitofusins and optic atrophy 1, determine the shape of organelles and segregation of damaged components for disposal [33]. AMPK activation, which occurs under energy stress that mimics ketosis, promotes fission through phosphorylation of the mitochondrial fission factor and simultaneously drives PTEN-induced kinase 1- and Parkin-dependent mitophagy, thereby clearing dysfunctional organelles [12]. In mouse C2C12 myotubes, medium-chain fatty acids, a component of the MCT diet, exert differential effects on these quality-control pathways and the maturation of the mitochondrial network, indicating that the composition of a ketogenic regimen affects organelle turnover [34]. In neurodegeneration models, restoring the balance of biogenesis, dynamics, and mitophagy is protective, and dietary strategies that increase BHB levels are being examined as a means of achieving this [33,35]. The relevance of these effects in patients remains largely inferential, as most data are derived from animal and cell models rather than from human tissues [12].

### 3.6. The Mitochondria–Inflammation Circuit

A further mechanism connects BHB to the innate immune system through direct inhibition of the NLR family pyrin domain-containing 3 (NLRP3) inflammasome, a sensor that activates caspase-1 and releases interleukin-1β and interleukin-18 [26]. In mouse macrophages, BHB, but not acetoacetate or the related short-chain fatty acids butyrate and acetate, suppresses NLRP3 activation in response to diverse danger signals, and does so specifically, without inhibiting the absent in melanoma 2 or NLR family CARD domain-containing protein 4 inflammasomes [36]. This selectivity distinguishes the action from a generic anti-inflammatory effect and points to a defined molecular target [30]. This mechanism is notable because it is independent of the metabolic and receptor pathways described above, as it does not require BHB oxidation in the TCA cycle and does not depend on HCAR2, AMPK, or sirtuins; instead, BHB prevents potassium efflux and blocks assembly of the inflammasome complex [36]. By reducing interleukin-1β and interleukin-18 secretion from human monocytes and attenuating inflammasome-mediated disease in mouse models, the finding extends toward human relevance [36,37].

The importance of this circuit, where mitochondrial damage and inflammation reinforce each other, is clear. In a mouse model of heart failure with a preserved ejection fraction, mitochondrial hyperacetylation promoted the assembly of the NLRP3 inflammasome on damaged mitochondria, driving cytokine release and fibrosis, whereas increasing BHB availability disrupted this cycle and rescued the phenotype [38]. Notably, BHB acts not by serving as an oxidative substrate but by reducing the mitochondrial acetyl-CoA pool and protein acetylation, again separating its signaling role from its role as a fuel [36,38]. Human data are beginning to accumulate, with one study reporting that a KD improves mitochondrial respiration in circulating monocytes and reduces lipopolysaccharide-driven signaling in people with obesity [37]. This suggests that the anti-inflammatory action of BHB is one of the few mechanisms with preliminary support in human cells rather than animal models alone [36,37].

### 3.7. Discordant Evidence and Boundary Conditions

The effects of ketosis on mitochondria are not uniformly beneficial, and several observations contradict the mechanisms described in this section. The clearest example is mitochondrial one-carbon metabolism, a pathway that generates formate for nucleotide synthesis and methylation. In HepG2 cells treated with physiological BHB and in mice fed an MCT KD, mitochondrial formate production was suppressed, plasma formate decreased by approximately 19%, and the flux of one-carbon units from serine and glycine was reduced [16]. This suppression of the biosynthetic pathway may contribute to adverse effects during prolonged ketogenic feeding, in contrast to the enhancement of oxidative pathways emphasized elsewhere [11,16]. The tissue specificity of ketone handling further limits generalization because the enzymes and transporters that make BHB useful in one organ are unevenly distributed and can be deficient in diseases [7].

**Figure 3:**
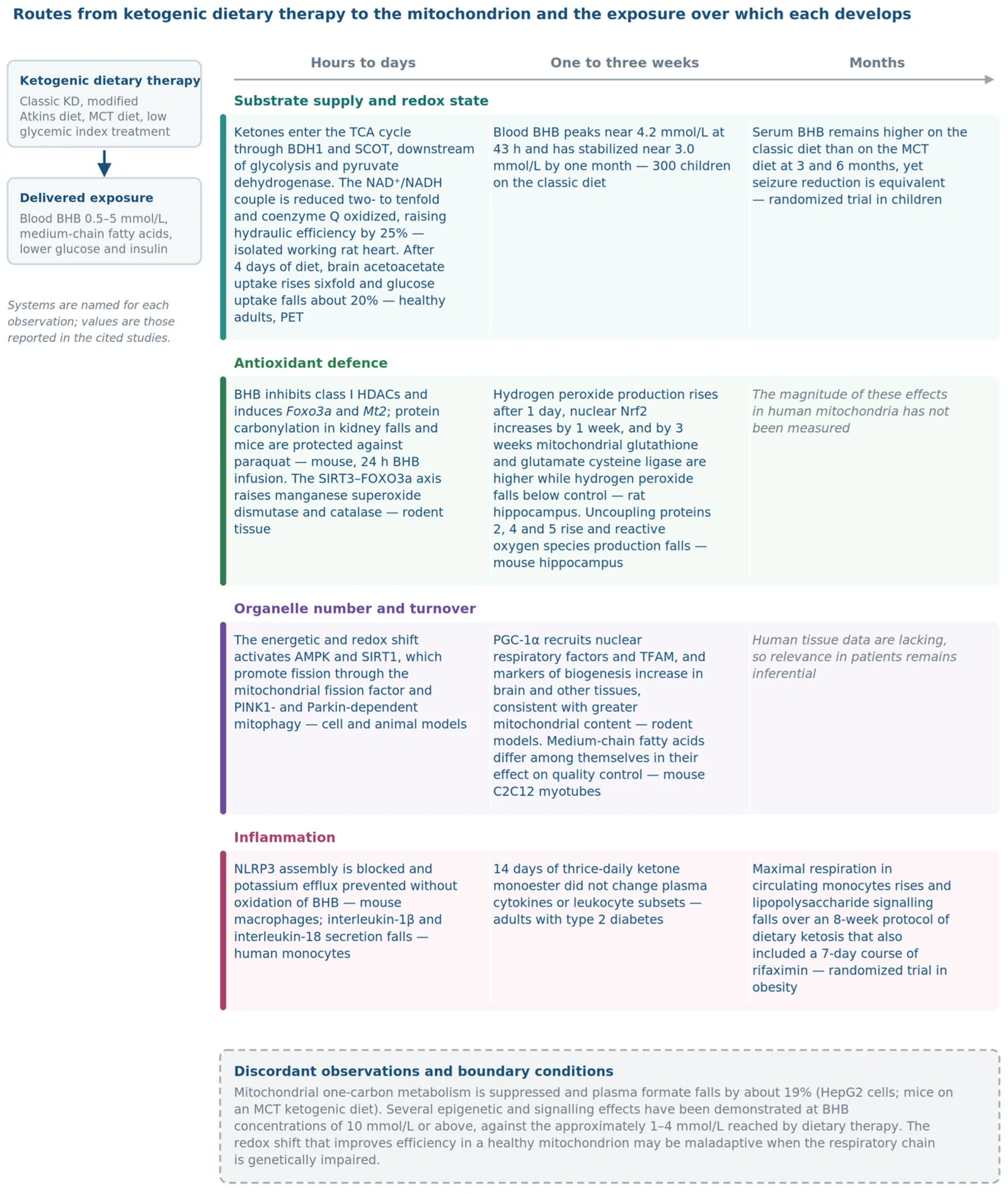
Routes from ketogenic dietary therapy to the mitochondrion and the exposure over which each develops. Values shown are those reported in the studies cited in the main text, and the experimental system is named for each observation because most mechanistic data derive from animal or cell models rather than from patients. Dietary therapy delivers BHB together with medium-chain fatty acids and a lower glucose and insulin concentration, and acts through four routes. Substrate supply and redox state change first, as ketones enter the TCA cycle downstream of glycolysis and pyruvate dehydrogenase and shift the NAD^+^/NADH and coenzyme Q couples. Antioxidant defense develops over days to weeks through induction of *Foxo3a* and *Mt2* after class I HDAC inhibition, through Nrf2-dependent glutathione synthesis that follows a transient early rise in hydrogen peroxide, through uncoupling proteins that limit electron leak, and through the SIRT3–FOXO3a axis. Organelle number and turnover change over weeks through AMPK and SIRT1 acting on PGC-1α and TFAM and through PINK1- and Parkin-dependent mitophagy. Inflammation is restrained within hours by inhibition of the NLRP3 inflammasome, an action that does not require oxidation of BHB. Cells marked in grey italics indicate where the corresponding measurement has not been made in patients. The dashed band lists the discordant and limiting observations discussed in Section 3.7. Gene names are italicized and protein names are in roman type. AMPK, AMP-activated protein kinase; BDH1, D-β-hydroxybutyrate dehydrogenase 1; BHB, β-hydroxybutyrate; HDAC, histone deacetylase; KD, ketogenic diet; MCT, medium-chain triglyceride; NAD, nicotinamide adenine dinucleotide; NLRP3, NLR family pyrin domain containing 3; Nrf2, nuclear factor erythroid 2-related factor 2; PET, positron emission tomography; PGC-1α, peroxisome proliferator-activated receptor gamma coactivator 1-alpha; PINK1, PTEN-induced kinase 1; SCOT, succinyl-CoA:3-oxoacid-CoA transferase; SIRT, sirtuin; TCA, tricarboxylic acid; TFAM, mitochondrial transcription factor A.

The duration of exposure is a further boundary condition, because the mechanisms described in this section do not develop on a single time scale (Figure 3). In rodents the antioxidant response is biphasic, as mitochondrial hydrogen peroxide production rises during the first days of feeding and falls below control values only after 3 weeks, by which time glutathione synthesis has been induced [11]. Most of the cellular data on BHB were obtained within minutes to hours, and the investigators of one such study noted that short-term signaling actions still need to be separated from longer-term effects on gene expression [23]. Inhibition of the NLRP3 inflammasome is demonstrable within hours of exposure in macrophages, whereas increases in mitochondrial content accumulate over weeks of feeding [11,36]. Human data are too sparse to define these intervals, since the improvement in monocyte respiration observed in people with obesity followed 8 weeks of dietary ketosis and the shortest effective exposure has not been determined [37]. Studies that compare defined durations of exposure, rather than reporting a single time point, are among the stated priorities for this field [6].

The concentration dependence of these mechanisms is a second boundary condition that can easily be overlooked (Table 1). Several epigenetic and signaling effects have been demonstrated at BHB concentrations of ≥10 mmol/L, well above the concentrations reached by dietary therapy, which range from approximately 1–2 mmol/L with the least ketogenic formats to approximately 3–4 mmol/L on the classic diet, raising questions about whether they operate at clinically relevant exposures [8,10]. The direction of the effect can also be reversed with context, as illustrated by the disputed agonist–antagonist behavior of BHB at FFAR3 [32]. Furthermore, the redox shift that improves efficiency in a healthy mitochondrion may be maladaptive when the respiratory chain is genetically impaired, which may explain why ketogenic therapy is contraindicated in specific mitochondrial and fatty acid oxidation disorders [14,15].

## 4. Achieving and Measuring Ketosis in Humans

These molecular mechanisms operate over a defined range of BHB concentrations; therefore, their clinical relevance depends on the level of ketosis that a strategy can actually produce. Nutritional ketosis is conventionally defined by a blood BHB concentration between approximately 0.5 and 5.0 mmol/L, well above the near-absent fasting baseline and well below the concentrations of diabetic ketoacidosis [39]. On the classic diet, the rise is rapid: in a cohort of 300 children initiated after fasting, BHB reached 2.0 mmol/L at 19 h, and among responders it peaked at 4.2 mmol/L at 43 h before stabilizing near 3.0 mmol/L from 1–3 months [40]. Fasting and sustained exercise raise BHB to the lower part of this range, near 1–2 mmol/L, while prolonged energy restriction leads to higher values [8]. The KD and its variants are calibrated to keep patients within this window, and consensus protocols specify how the diet is initiated and titrated to maintain it [2]. Since the effects of BHB are concentration-dependent, the practical question is not whether a strategy induces ketosis but how high, how quickly, and how consistently it raises BHB [7].

Dietary formats differ mainly in the carbohydrate restriction they require and the steadiness of ketosis they produce (Table 2). The KD, delivered at a 4:1 or 3:1 ratio of fat to combined protein and carbohydrates, produces the most sustained ketosis but is the most restrictive, whereas the MAD and MCT diets achieve comparable seizure control with greater flexibility [15]. In the only randomized comparison of the classic and MCT diets, serum BHB was significantly higher in the classic diet at 3 and 6 months, yet the two produced equivalent seizure reductions, showing that a lower average ketone level does not necessarily indicate a weaker clinical effect [41]. LGIT is the least restrictive and generally produces the lowest and most variable BHB; accordingly, it is not recommended for glucose transporter type 1 deficiency syndrome or pyruvate dehydrogenase complex deficiency, where adequate ketosis is essential [2]. MCTs are absorbed and oxidized more rapidly than long-chain fats; therefore, the MCT diet tends to produce a postprandial peak in ketones rather than the flatter profile of the classic diet [7]. These differences in the shape of the ketone curve, in addition to its average concentration, are likely to affect mechanisms that depend on sustained exposure or transient peaks [8].

Within the MCT format, the individual medium-chain fatty acids are not interchangeable, and formulations enriched in decanoic acid have been developed on that basis [6]. Caprylic and capric acids act differently on mitochondrial quality control and on respiration in myotubes, so the fatty acid composition of a regimen, and not only the ketone concentration it produces, bears on the organelle [34]. A medical food composed principally of decanoic acid has been trialed in drug-resistant epilepsy, where the proposed benefit combines an effect on energy metabolism with an effect on network excitability and better palatability, and decanoic acid additionally inhibits mechanistic target of rapamycin complex 1 in ex vivo rat hippocampus and in astrocytes derived from patients with tuberous sclerosis complex [6]. These actions do not require ketosis, which is consistent with the randomized finding that the MCT diet produces lower serum BHB than the classic diet without a smaller seizure reduction [41]. Blood BHB therefore describes only part of the exposure delivered by an MCT-based regimen, since the fatty acids themselves are absorbed and oxidized rapidly and act on targets that ketone measurement does not capture [7].

Ketosis can also be pharmacologically induced by uncoupling BHB exposure from carbohydrate restriction, which allows the ketone level to be titrated directly. The ketone monoester (R)-3-hydroxybutyl (R)-3-hydroxybutyrate raises plasma BHB to approximately 3.3 mmol/L within 1–2 h in healthy adults, with an elimination half-life of 0.8–3.1 h, and a comparable drink can raise blood D-BHB from near zero to roughly 3.5 mmol/L within 10 min without dietary change [10,42]. Ketone salts provide an alternative route; a mixture of sodium, calcium, and magnesium D-BHB salts achieved a peak of approximately 1.2 mmol/L within 1 h in healthy adults and were tolerated in children with inherited metabolic disorders for up to 2 years and 7 months [43]. The isomeric form is important because only the physiological D-isomer is efficiently used as fuel [17]. Preparations delivering the pure D-form achieved 1.5- to 2-fold higher effective concentrations than racemic D, L salts at equal doses [43]. These exogenous strategies produce briefer, shallower ketosis than a maintained diet but make BHB a titratable variable and are being examined when dietary adherence is impractical [10,42].

Regardless of the route, the value of ketosis in practice depends on the ability to measure it accurately, and the evidence strongly favors one method. Capillary blood BHB level, measured using a point-of-care meter, is the current reference standard for assessing nutritional ketosis, and it can replace laboratory assays when required [39]. In contrast, urinary testing reflects acetoacetate and correlates poorly with blood ketosis, as a strongly positive urine dipstick corresponds to a wide range of blood BHB levels, from approximately 2–12 mmol/L [44]. Whether a specific blood BHB level predicts seizure control is itself contested: one early study found that children with BHB level >4 mmol/L were more likely to respond, whereas a recent cohort of 300 children found no difference in BHB levels between responders and non-responders [40,44]. Because a single BHB value is an imperfect predictor and cannot distinguish a genuine metabolic shift from a transient change, the glucose–ketone index, calculated as the ratio of blood glucose to BHB in millimoles per liter, is increasingly used to express the overall metabolic state in one figure [7]. Developed to monitor therapeutic ketosis in oncology, and now applied across neurological and psychiatric indications, this composite provides a more informative target than BHB level alone [39].

Point-in-time testing also misses the substantial fluctuations in BHB level across the day, which emerging technologies are beginning to address. Continuous ketone monitors and wearable sensors that sample the interstitial fluid provide real-time data and can reveal circadian and behavioral patterns in ketone production that intermittent testing misses [39]. Such devices are relevant given the concentration-dependent action of BHB, as matching a molecular effect to an achievable exposure requires a full concentration–time profile rather than a single reading [10]. Their accuracy and clinical validation have not yet been established; therefore, blood BHB remains the practical standard [39]. Reliable, ideally continuous measurement is therefore a prerequisite for determining whether these mechanisms operate at the actual exposures that patients attain [42].

## 5. Clinical Translation Across Disease Domains

### 5.1. Drug-Resistant Epilepsy

Drug-resistant epilepsy is defined as persistent seizures despite adequate trials with two tolerated and appropriately chosen anti-seizure medications [45]. It affects approximately 30% of patients with epilepsy, and approximately 48% of those treated with KDTs achieve at least 50% seizure reduction or seizure freedom (Table 3) [6]. The first randomized trial established the effect size, with 38% of children assigned to the diet achieving >50% seizure reduction at 3 months compared with 6% of controls [5]. A subsequent network meta-analysis confirmed that the KD, MAD, and LGIT are superior to usual care for achieving at least 50% seizure reduction [3]. In a three-arm randomized trial of 158 children who completed 24 weeks of treatment, the median seizure reduction was 66% with the KD, 45% with the MAD, and 54% with LGIT; although neither variant met the predefined non-inferiority margin, treatment-related adverse events were markedly fewer with LGIT than with the two other diets (33.3% vs. 56.4% and 56.9%) [4]. The MAD also outperformed levetiracetam in a separate randomized trial, in which 52.9% versus 22% of children achieved at least a 50% reduction in seizures [6].

Each variant was developed to relax the specific constraints of the classic diet. The MCT diet was devised because severe carbohydrate restriction makes the classic regimen unpalatable, and it was first tested in 12 children and adolescents aged 2.5–16 years with previously intractable seizures [46]. The Atkins approach was introduced with the same rationale, removing calorie and protein restrictions, and in six patients aged 7–52 years, five maintained moderate to large ketosis, and three had seizure reduction sufficient to reduce medication [47]. LGIT went further by liberalizing total carbohydrates to foods with a glycemic index <50, and 10 of 20 treated patients had >90% seizure reduction, even though this regimen produced less ketosis than the classic diet [11,48]. Initiation was simplified in parallel, since a nonfasting protocol in 41 children produced 34.1% seizure freedom compared with 34.9% after conventional fasting, whereas moderate dehydration decreased from 62.7% to 12.2% [49]. These formats are now delivered under consensus protocols that specify patient selection, initiation, supplementation, and laboratory monitoring [2]. Evidence in adults remains considerably thinner, with a single randomized comparison of KDT formats rated as having very low certainty, whereas the MAD has proven feasible in low- and middle-income settings where dietitian support is limited [6]. Genotypes have been proposed as additional criteria for selecting candidates and the appropriate diet format [15].

Adverse effects of dietary therapy are common but seldom serious, and their timing differs with the duration of treatment [2]. Gastrointestinal symptoms dominate the first weeks, and in the randomized comparison of the classic and MCT diets constipation was reported by a third to a half of children at every assessment over 12 months, with lack of energy more frequent on the classic diet at 3 months and vomiting at 12 months [41]. Hyperlipidemia is reported in 14–59% of children on the classic diet, can appear as early as the first month, and usually returns towards baseline by 12 months, whereas renal calculi occur in 3–7% and are largely prevented by oral citrates [2]. Gastrointestinal symptoms and abnormal lipid profiles are likewise the most frequent adverse events in adults, in whom they contribute to discontinuation [6]. With longer treatment, height deceleration, reduced bone mineral density, and secondary carnitine and selenium deficiency become the principal concerns, and cardiomyopathy and prolonged QT interval have been reported rarely, which is why consensus protocols specify metabolic screening before initiation and scheduled monitoring of lipids, carnitine, selenium, urine calcium, and bone mineral density [2].

### 5.2. Primary Mitochondrial Disease and Mitochondrial Epilepsy

Respiratory-chain disorders are under dual genetic control, and heteroplasmy together with a threshold effect explains why identical mutations produce tissue-specific phenotypes [67]. The minimum birth prevalence of mitochondrial disease is approximately 20 cases per 100,000 individuals in most regions worldwide [13]. Epilepsy occurs in 20–50% of patients over the disease course and in 35–60% of pediatric patients, and pathogenic variants in DNA polymerase gamma (POLG) are the most frequent single genetic cause at all ages [68]. Respiratory-chain defects also account for a substantial proportion of epilepsies that are otherwise unexplained [69]. Ketone oxidation supplies acetyl-CoA to the TCA cycle without passing it through glycolysis, and complex I disorders are among the conditions in which the KDT response consistently exceeds the average dietary response [2,11]. Pharmacological options are simultaneously constrained because valproate must be avoided in *POLG*-related diseases owing to the risk of hepatic failure and seizure aggravation [68,70].

A single-center study first indicated that the KD can be delivered safely and effectively in children with epilepsy and mitochondrial respiratory chain complex defects, including those in whom the defect was identified as the cause of previously unexplained epilepsy [69,71]. Controlled data were obtained from a multicenter open-label study of 33 patients with genetically confirmed disease, in which 40.9% of the 22 patients assigned to the diet achieved at least a 50% reduction in seizures at 3 months, whereas among the 23 participants who completed the protocol the response rate was 90% in mitochondrial encephalomyopathy, lactic acidosis, and stroke-like episodes (MELAS) and 93.3% in carriers of pathogenic mitochondrial DNA (mtDNA) variants [50]. The response rate among completers carrying nuclear DNA variants was 37.5%, and a 2021 systematic review that identified only 20 genetically confirmed cases from 694 publications reported seizure control in seven of eight [14]. A mechanistic counterpart exists for the MELAS phenotype, as adding ketone bodies to a MELAS neuronal cybrid cell model restored the assembly and stability of complex I [72]. Clinical reports point in the same direction, describing improved seizure control, fewer stroke-like episodes after adjunctive dietary therapy, and progressive resolution of headache, aphasia, and visual aura in adults with *POLG*-related epilepsy treated with LGIT [73,74]. Because the classic diet has been difficult to sustain long term, MAD and LGIT are increasingly offered in this population, where they reduce seizure frequency and lactate burden with fewer gastrointestinal and metabolic effects [75].

KDTs are contraindicated in disorders that depend on intact fatty acid oxidation, including primary carnitine deficiency, carnitine palmitoyltransferase I and II deficiency, carnitine translocase deficiency, defects of medium-, long-, and short-chain acyl-CoA dehydrogenase and of long-chain 3-hydroxyacyl-CoA dehydrogenase, together with pyruvate carboxylase deficiency and porphyria [2,6]. Pathogenic *SLC22A5* variants cause primary carnitine deficiency, and pyruvate carboxylase deficiency impairs the anaplerotic replenishment of oxaloacetate, leaving patients dependent on glucose and vulnerable to metabolic acidosis when fat oxidation is forced [15]. Within mitochondrial disease, the risk is genotype-specific rather than general, and all five adults with mtDNA deletion-related myopathy who began MAD discontinued it within 4–11 days because of rhabdomyolysis, headache, and fatigue [76]. Three individuals with *POLG*-related Alpers syndrome died while receiving the diet, although their survival did not differ from that of patients with *POLG* variants who were not treated [14]. Hepatic dysfunction is the principal concern in *POLG*-related diseases. LGIT, which permits partial glucose availability, has been proposed as a safer option for patients with mtDNA deletions [15]. Accordingly, KDT delivery requires an experienced team, baseline metabolic screening, and scheduled monitoring of lactate, liver function, and carnitine and selenium levels, consistent with the published care standards for primary mitochondrial disease [2,77].

The position of a defect within the respiratory chain, rather than its severity, determines whether ketone oxidation can compensate for it. Ketone bodies enter the TCA cycle downstream of glycolysis and the pyruvate dehydrogenase complex, but the reducing equivalents they generate reach coenzyme Q through complex I, as do those released by β-oxidation through the electron-transfer flavoprotein and its dehydrogenase, so a defect at the level of coenzyme Q is not bypassed [67]. The gain in efficiency attributed to ketones in fact presupposes an intact pool, since it arises from oxidation of the coenzyme Q couple and the widened redox span that follows [22]. A ketogenic regimen may also increase the demand on this step by shifting the tissue towards fat oxidation, which is the reason disorders of fatty acid transport and oxidation are absolute contraindications to dietary therapy [2]. Exogenous ketone bodies do not carry that requirement and have been given long term to children with multiple acyl-CoA dehydrogenase deficiency, a disorder of the same electron-transfer step, and with pyruvate dehydrogenase deficiency [43]. Direct clinical experience is limited to a few patients, since in the multicenter trial described above two participants carried *COQ4* variants, of whom one completed the protocol with at least a 50% reduction in seizures, whereas an infant with primary coenzyme Q10 deficiency-7 showed marked seizure improvement but died one month after starting the diet from a cause the investigators could not determine [50]. These few observations are insufficient to judge either efficacy or safety in this group, and a 2021 systematic review of the literature on dietary therapy in mitochondrial disease identified only 20 genetically confirmed cases [14].

### 5.3. Neurodegenerative and Psychiatric Disorders

Mitochondrial dysfunction is an early and consistent feature of neurodegenerative disease, with reduced complex I activity, impaired mitophagy, and accumulated oxidative damage documented in Alzheimer’s disease (AD) and Parkinson’s disease [35]. Cerebral glucose hypometabolism appears years before symptoms and is more pronounced in carriers of the *APOE4* allele, whereas brain ketone uptake remains proportional to plasma concentration, providing a metabolic rationale for ketone supplementation as an alternative fuel [21,24]. The largest controlled test administered 20 g of MCT daily for 90 days to 152 patients with mild to moderate AD. Those without the APOE4 allele showed greater improvement in the AD Assessment Scale–cognitive subscale than placebo recipients, with post-dose serum BHB correlating positively with improvement [11,51]. The same genotype dependence was observed in a crossover trial of 49 patients given 17.3 g MCT daily for 4 weeks, in which cognition improved only in *APOE4*-negative participants [52]. Longer exposure produced broader gains, as a ketogenic drink taken twice daily for 6 months by 83 patients with mild cognitive impairment improved episodic memory, language, and executive function [53]. A modified Mediterranean KD in adults at risk of AD improved their cerebrospinal fluid biomarker profile, cerebral perfusion, and cerebral ketone uptake [54].

Evidence in Parkinson’s disease is smaller in scale but consistent in direction, beginning with a feasibility study in which seven patients sustained ketosis for 28 days with improvement in motor scores [55]. In a randomized parallel-group comparison of a KD with a low-fat diet in 47 patients over 8 weeks, both groups improved in motor and nonmotor symptoms, but the advantage of the ketogenic arm was confined to nonmotor symptoms, with no difference between the groups in the motor domains [24]. The same mitochondrial abnormalities have been described in psychiatric diseases, including reduced complex I activity and altered expression of nuclear-encoded mitochondrial genes in patients with schizophrenia and bipolar disorder [78]. A 4-month pilot trial of a diet providing 60% fat, 30% protein, and 10% carbohydrates to patients with schizophrenia or bipolar disorder found a 32% reduction in Brief Psychiatric Rating Scale scores among those with schizophrenia, together with improvement in metabolic parameters [26,56]. A separate pilot study in bipolar disorder found that the diet was acceptable and deliverable in an outpatient setting [57]. The proposed mediators include replenishment of oxaloacetate, which shifts glutamate metabolism toward GABA synthesis, and induction of brain-derived neurotrophic factor through HDAC2 and HDAC3 inhibition, a link supported in humans by the association between serum pro-BDNF and changes in blood BHB levels [26,79].

Trials in these populations remain small, short, and heterogeneous in the intervention delivered, and most report cognitive or symptom scales rather than disease progression [24]. Long-term safety is less well-characterized here than in epilepsy, and adherence is the principal practical constraint for interpreting negative results [26]. Dietary therapy has also been applied to neurodevelopmental conditions, with a pilot study of KD in children with autistic behavior and a later trial of a modified ketogenic gluten-free diet containing MCT, both reporting behavioral improvement [80,81]. These indications overlap with those of epilepsy, in which cognitive and behavioral comorbidities are frequent and may respond to the same intervention [82]. Whether the benefit reflects restored mitochondrial bioenergetics, epigenetic signaling, or reduced neuroinflammation has not been determined in human studies because routine practice measures only blood BHB as a marker of exposure [35,39].

### 5.4. Cardiometabolic Disease

The heart consumes more ketone bodies per unit mass than any other organ and oxidizes them in proportion to delivery at the expense of fatty acid and glucose oxidation [7]. Quantitative mitochondrial proteomics in mouse models showed that proteins involved in fatty acid utilization decreased, whereas BDH1 increased, and isolated heart preparations confirmed a shift toward ketone oxidation once the capacity to oxidize fatty acids was reduced [83]. The same reprogramming occurs in patients, since myocardium from nondiabetic individuals with advanced heart failure showed depleted long-chain acylcarnitines, accumulated acetyl-CoA, increased β-hydroxybutyryl-CoA, and higher *OXCT1* expression [84]. Preventing 3-hydroxybutyrate oxidation in the heart worsened heart failure after fasting or pressure overload, whereas increased ketone delivery improved remodeling and function in mice and a canine pacing model [85]. The corresponding human experiment raised plasma 3-hydroxybutyrate from 0.4 to 3.3 mmol/L by infusion in 16 patients with reduced ejection fraction and increased cardiac output by 2.0 L/min through a 20 mL rise in stroke volume, with a measurable effect from 0.7 mmol/L upward and no loss of myocardial external efficiency [58]. In heart failure with a preserved ejection fraction, increasing BHB with a ketone ester or with the sodium-glucose cotransporter 2 inhibitor empagliflozin lowered mitochondrial protein acetylation and restored complex I- and complex II-supported respiration in mice [38]. Sodium-glucose cotransporter 2 inhibition also increases circulating ketone concentrations in humans, although its contribution to the clinical benefits of these agents remains unclear [7].

In type 2 diabetes, a non-randomized continuous-care program delivering nutritional ketosis lowered glycated hemoglobin from 7.6% to 6.3% and body weight by 13.8 kg over 1 year in 262 adults, with insulin reduced or discontinued in 94% of users [59]. Among the 122 participants followed up for 5 years, 20% achieved remission and 32.5% had glycated hemoglobin <6.5% without glucose-lowering medication or metformin alone and with sustained reductions in body mass and triglycerides and no significant change in low-density lipoprotein cholesterol [60]. Randomized evidence is less consistent since a crossover trial in 33 adults with prediabetes or type 2 diabetes found no difference in glycated hemoglobin between a well-formulated KD and a Mediterranean-style diet after 12 weeks each, along with a 10% increase in low-density lipoprotein cholesterol in the ketogenic arm [61]. Hepatic effects are better defined mechanistically, since 6 days of diet in ten overweight adults reduced intrahepatic triglyceride levels by 31% and hepatic insulin resistance by 58%, changes attributed to a 38% decrease in hepatic citrate synthase flux and a 167% rise in hepatic mitochondrial redox state that redirected fatty acid carbon toward ketogenesis [62]. Anti-inflammatory effects are harder to demonstrate in patients, because 14 days of thrice-daily ketone monoester in type 2 diabetes did not alter plasma cytokines or leukocyte subsets even though direct exposure of leukocytes to BHB suppressed interleukin-1β, tumor necrosis factor-α, and interleukin-6 [63]. Sustained dietary ketosis improves mitochondrial respiration in circulating monocytes in people with obesity, although that trial also included a 7-day course of rifaximin between the two dietary periods [37].

### 5.5. Cancer

Tumor cells sustain a high glycolytic flux even under aerobic conditions, and dietary ketosis has been proposed to exploit this dependence by lowering glucose availability [7]. Multiomic profiling of livers from mice on the diet mapped 5304 lysine β-hydroxybutyrylation sites and showed that modification of aldolase B at Lys108 inhibits mammalian target of rapamycin signaling and suppresses proliferation and xenograft growth in hepatocellular carcinoma cells [29]. A second pathway operates through the receptor route, as, in mice, BHB reduces colonic crypt proliferation and suppresses intestinal tumor growth by acting on HCAR2 and inducing the transcriptional regulator HOP homeobox. Organoid assays, together with single-cell sequencing of patient biopsies, linked higher BHB and active HOP homeobox levels to reduced intestinal epithelial proliferation in humans [86]. Clinical data remain limited to small trials, and among 80 patients with locally advanced or metastatic breast cancer randomized for 12 weeks, quality of life and physical activity did not differ between the groups at the end of the study, although serum lactate and alkaline phosphatase levels decreased in the ketogenic arm [64]. In rectal cancer patients treated with radiochemotherapy, 18 patients on the diet maintained role, emotional, and social functioning, while 23 on a standard diet declined, yet approximately half described daily implementation as difficult [65]. A prospective glioblastoma study reported 3-year survival in four of six patients who sustained the diet beyond 6 months compared with 8.3% of those who did not, a difference that is difficult to interpret because adherence may indicate a better prognosis [66]. A meta-analysis has linked the survival benefit to the depth of ketosis, timing of initiation, and tumor location, whereas a rat glioma model showed no effect on tumor volume or survival despite increased BHB [7].

## 6. Unresolved Questions in Clinical Translation

The mechanisms described above have been established almost entirely in cells and animals, and very few human trials have directly measured the mitochondrial outcomes. Hepatic mitochondrial fluxes and redox states were quantified by stable isotope tracer analysis in ten adults after 6 days of feeding [62]. Oxygen consumption in circulating monocytes was measured in a randomized trial in people with obesity, and these two studies provide some of the closest available evidence in humans [37]. Elsewhere, the mitochondrial contribution is inferred from the clinical outcome; therefore, the specific contribution of each of the four routes described above cannot be determined [35,39]. Blood BHB is the only mediator measured in routine practice, and its relationship to outcome is not a simple dose–response since the classic diet produces higher concentrations than the MCT diet without greater seizure reduction [41]. The same holds for LGIT, which produces the lowest and most variable ketosis of the dietary formats yet allowed 10 of 20 treated patients to achieve >90% seizure reduction [2,48].

Several features of the existing trials also limit the extent to which their results can be extended. Exogenous ketones and dietary ketosis are not equivalent because an ester raises BHB within minutes while glucose and insulin remain relatively unchanged, whereas dietary ketosis lowers both over days, and racemic salts supply only the D-enantiomer as a substrate [10,43]. This distinction may explain why 14 days of ketone monoester treatment left plasma cytokines and leukocyte subsets unchanged in type 2 diabetes [63]. Genotype has repeatedly modified treatment response, with cognitive benefits confined to *APOE4*-negative participants in MCT trials [52]. The response also appears concentrated among carriers of mtDNA variants in mitochondrial disease, whereas *POLG*-related disease carries a risk of harm; however, no trial has prospectively stratified patients by genotype [15,50]. Trials outside of epilepsy have been conducted for weeks to months, leaving long-term safety in psychiatric and neurodegenerative populations unestablished [26]. Adherence may itself mark a better prognosis; therefore, differences in survival and quality of life in non-randomized oncology cohorts cannot be attributed to diet alone [65,66].

## 7. Future Directions

Genotype already influences the decision to prescribe dietary therapy, as glucose transporter type 1 deficiency syndrome and pyruvate dehydrogenase complex deficiency are absolute indications, while variants in pyruvate carboxylase and *SLC22A5* are contraindications [15]. Extending that logic to probabilistic prediction is the next step because the response in mitochondrial disease is concentrated in carriers of mtDNA variants and the cognitive benefit in trials of MCT has been confined to *APOE4*-negative participants [50]. Genome-wide association and multiomic studies have begun to identify metabolic pathways that are associated with the response to a classic diet, and the gut microbial composition is being examined alongside them [15]. Validating such candidates across several epilepsy syndromes is a stated research priority, together with standardizing protocols for initiation and adherence [6]. What is missing is a trial that prospectively assigns therapy according to genotype and reports outcomes by stratum rather than recovering the association afterward [52].

Monitoring will also have to be richer than a single fasting value if the concentration–response relationship is to be defined. Continuous ketone monitors measure interstitial fluid through a subcutaneous sensor, and a first-in-human study in 12 participants found 82.4% of paired readings within 0.225 mmol/L or 20% of the capillary reference over 14 days [87]. A consensus panel of 20 clinicians and scientists adopted 30 conclusions regarding this technology, noting that accuracy and calibration require validation [88]. Breath acetone offers a noninvasive alternative for the population most burdened by repeated sampling since it closely tracked plasma BHB in children on the diet, although it did not predict seizure frequency [89]. The glucose–ketone index was introduced for metabolic management of brain cancer and has since been applied more broadly [39]. Whether any of these tools improve outcomes rather than simply documenting adherence has not yet been tested [40].

Several mechanistic questions can be answered using existing methods. Stable isotope tracer studies and respirometry in accessible cells have been performed in small human cohorts, and applying them in randomized trials would show whether mitochondrial changes are associated with clinical benefits [37]. Germ-free mice do not respond to the diet unless colonized by a microbiome from a responding animal, suggesting that the gut microbiome may mediate part of the response and supporting the evaluation of microbiome-directed adjuncts [90]. Ketone esters increase BHB without carbohydrate restriction, thus separating the contribution of the metabolite from that of the diet [10,43]. In mitochondrial diseases, the most informative trial would stratify participants by genotype at entry, given the divergence between mtDNA- and *POLG*-related disease [6].

## 8. Conclusions

BHB is a respiratory fuel that enters the TCA cycle downstream of glycolysis, and it is also a signal acting on histone modification, surface receptors, and mitochondrial quality control [7]. The clinical record is strongest when the evidence is most established, with randomized trials supporting KDTs in drug-resistant epilepsy and observational series showing genotype-dependent benefits in primary mitochondrial disease [6,50]. Outside neurology, human data comprise a hemodynamic response to infused 3-hydroxybutyrate in heart failure and sustained glycemic improvement in non-randomized diabetes cohorts [58,60]. Few studies have measured a mitochondrial endpoint directly; therefore, the mechanisms reviewed here remain plausible rather than a demonstrated explanation of the clinical effect [37]. Genotype-stratified trials, more comprehensive exposure monitoring, and direct measurement of mitochondrial variables in patients are the most likely steps to establish which effects are real and in whom [15].

## Figures and Tables

**Table 1:** Molecular actions of β-hydroxybutyrate on mitochondrial function, with the concentration at which each was demonstrated and the highest level of evidence available.

Action	Molecular Target or Readout	Concentration Demonstrated	Highest-Level System	Ref.
**Bioenergetics and redox**				
Increased thermodynamic efficiency	Hydraulic efficiency +25%; +36% with insulin	Physiological ketone ratio	Isolated working rat heart	[22]
Widened redox span	Mitochondrial NAD^+^/NADH reduced 2- to 10-fold; coenzyme Q oxidized	Physiological ketone ratio	Isolated working rat heart	[7,22]
Greater ATP-linked respiration	Higher ATP-coupled oxygen consumption; sustained maximal uncoupled respiration	5 mmol/L	Primary mouse cortical neurons	[23]
Substrate entry downstream of glycolysis	Acetyl-CoA supply for oxidation	Physiological	Inferred from human data	[7,11]
**Oxidative stress defense**				
Reduced electron leak	Uncoupling proteins; more oxidized quinone pool	Dietary ketosis	Rodent tissue	[11,22]
Antioxidant transcriptional program	SIRT3–FOXO3a axis; manganese superoxide dismutase; catalase	Dietary ketosis	Rodent tissue	[24,25,26]
Protection against oxidant injury	*Foxo3a* and *Mt2* induction; reduced renal protein carbonylation after paraquat	Exogenous BHB in vivo	Mouse	[8]
**Epigenetic regulation**				
Class I and IIa HDAC inhibition	HDAC1 and HDAC3 (class I); HDAC4 (class IIa); not HDAC6 (class IIb)	IC_50_ 5.3, 2.4, and 4.5 mmol/L; onset 1–2 mmol/L	Purified enzymes; mouse brain in vivo	[8,9,27]
Histone β-hydroxybutyrylation	44 Kbhb sites; H3K9bhb at active promoters	Proportional to circulating BHB	Human cells and mouse liver	[25,28]
Non-histone Kbhb	TP53; aldolase B Lys108 → mTOR suppression	Diet-induced	Mouse; human cancer cell lines	[25,29]
GABAergic shift	H3K27 acetylation; *Sirt4* and *Gad1* up-regulation; higher GABA/glutamate ratio	Diet-derived BHB	Mouse pentylenetetrazol model	[27]
**Surface receptor signaling**				
HCAR2 agonism	EC_50_ approximately 0.7 mmol/L; neuroprotection lost in *Hcar2*-deficient mice	Approximately 0.7 mmol/L (physiological)	Mouse stroke model	[9,31]
FFAR3 antagonism	Suppressed propionate-induced ERK signaling and heart rate rise	Physiological	Mouse sympathetic neurons	[32]
FFAR3 direction disputed	Agonism with *N*-type calcium channel inhibition reported	Physiological	Rat neurons	[30]
**Mitochondrial quality control**				
Biogenesis	AMPK and SIRT1 → PGC-1α → TFAM	Dietary ketosis	Rodent brain and tissue	[11,12,33]
Dynamics and mitophagy	AMPK → mitochondrial fission factor; PINK1/Parkin	Dietary ketosis	Cell and animal models	[12,33]
Regimen-dependent effects	Differential action of medium-chain fatty acids	Dietary MCT	Mouse C2C12 myotubes	[34]
**Inflammation**				
NLRP3 inhibition	Blocked potassium efflux and complex assembly; AIM2 and NLRC4 unaffected; independent of oxidation, HCAR2, AMPK, and sirtuins	Physiological	Human monocytes; mouse disease models	[36]
Mitochondria–inflammation circuit	Reduced mitochondrial acetyl-CoA pool and protein acetylation	Ketone ester or empagliflozin	Mouse HFpEF model	[38]
Human counterpart	Improved monocyte respiration; reduced lipopolysaccharide-driven signaling	Dietary ketosis	Randomized trial in obesity	[37]
**Discordant action**				
One-carbon metabolism suppressed	Mitochondrial formate production reduced; plasma formate −19%	Physiological	HepG2 cells; mice on MCT diet	[16]
Concentration gap	Several epigenetic and signaling effects shown at ≥10 mmol/L versus approximately 1–4 mmol/L achieved by dietary therapy	≥10 mmol/L	Cell and animal models	[8,10]

Abbreviations: AMPK, AMP-activated protein kinase; BHB, β-hydroxybutyrate; EC_50_, half-maximal effective concentration; ERK, extracellular signal-regulated kinase; FFAR3, free fatty acid receptor 3; GABA, γ-aminobutyric acid; HCAR2, hydroxycarboxylic acid receptor 2; HDAC, histone deacetylase; HFpEF, heart failure with preserved ejection fraction; IC_50_, median inhibitory concentration; Kbhb, lysine β-hydroxybutyrylation; MCT, medium-chain triglyceride; mTOR, mammalian target of rapamycin; NLRP3, NLR family pyrin domain containing 3; PGC-1α, peroxisome proliferator-activated receptor gamma coactivator 1-alpha; TFAM, mitochondrial transcription factor A.

**Table 2:** Strategies for inducing ketosis in humans and the resulting β-hydroxybutyrate exposure.

Strategy	Achieved Blood BHB (mmol/L)	Kinetic Profile	Level of Human Evidence	Principal Limitation	Ref.
Classic KD (4:1/3:1)	Peak approximately 4.2 (43 h); steady approximately 3.0 at 3 mo; reference range 1.1–4.9	Rapid rise, then sustained	Large cohort; RCT	Restrictive; low adherence	[40,41]
Modified Atkins diet	Generally lower than cKD; not systematically reported	Sustained	RCT	Slightly lower ceiling than cKD	[2,4]
MCT diet	2.71 ± 1.70 (vs. 4.21 ± 1.73 on cKD at 3 mo)	Postprandial peaks	RCT	GI intolerance	[41]
LGIT	Lowest, variable	Low, fluctuating	RCT	Shallow ketosis	[2]
Ketone monoester	up to approximately 3.3 (714 mg/kg)	Acute peak, t½ 0.8–3.1 h	Healthy-adult PK	Taste; GI at high dose	[10,42]
Ketone salt (D-βHB, e.g., NHS101)	Approximately 1.2 (Cmax, healthy adults)	Acute peak, 3–4 h	Case series, PK	Cation load; modest peak	[43]
Fasting/prolonged exercise	1–2 (higher with prolonged fast)	Slow rise	Physiology studies	Not a durable therapy	[8]

Concentrations are indicative values from cited human studies and vary with protocol, age, and adherence. Abbreviations: BHB, β-hydroxybutyrate; KD, ketogenic diet; cKD, classic ketogenic diet; MCT, medium-chain triglyceride; LGIT, low glycemic index treatment; RCT, randomized controlled trial; PK, pharmacokinetic; GI, gastrointestinal; t½, elimination half-life; Cmax, maximum concentration; mo, month.

**Table 3:** Human evidence for ketogenic dietary therapies across disease domains.

Domain	Intervention (Duration)	Population (*n*)	Design	Principal Human Outcome	Ref.
**Drug-resistant epilepsy**	cKD (3 mo)	Children (145)	RCT	≥50% seizure reduction in 38% vs. 6% of controls	[5]
	cKD, MAD, LGIT (24 wk)	Children (158 completers)	3-arm RCT	Median reduction 66%, 45%, 54%; treatment-related AEs 56.4%, 56.9%, 33.3%	[4]
	cKD, MAD, LGIT	Children	Network meta-analysis	Each superior to usual care for ≥50% reduction	[3]
	MCT diet	Children/adolescents 2.5–16 y (12)	Open label	First trial of the MCT format in intractable seizures	[46]
	MAD	Patients 7–52 y (6)	Open label	Five maintained moderate–large ketosis; three reduced medication	[47]
	LGIT	Patients (20)	Retrospective	>90% reduction in 10/20 with less ketosis than the classic diet	[48]
	Nonfasting initiation	Children (41)	Comparative	Seizure freedom 34.1% vs. 34.9%; dehydration 12.2% vs. 62.7%	[49]
**Primary mitochondrial disease**	cKD (3 mo)	Genetically confirmed (33)	Multicenter open-labelled controlled	≥50% reduction in 40.9% of the diet arm (9/22); among completers, MELAS 9/10 and mtDNA carriers 14/15	[50]
	KDTs	Genetically confirmed (20 cases from 694 publications)	Systematic review	Seizure control in 7/8; all five adults with mtDNA deletion myopathy stopped MAD within 4–11 d	[14]
**Alzheimer’s disease/MCI**	MCT 20 g/d (90 d)	Mild–moderate AD (152)	RCT	ADAS-cog improvement in *APOE4*-negative participants; BHB correlated with gain	[11,51]
	MCT 17.3 g/d (4 wk)	AD (49)	Crossover	Cognitive improvement only in *APOE4*-negative participants	[52]
	Ketogenic drink twice daily (6 mo)	MCI (83)	RCT	Improved episodic memory, language, and executive function	[53]
	Modified Mediterranean KD	Adults at risk of AD	Crossover	Improved CSF biomarker profile, cerebral perfusion, and ketone uptake	[54]
**Parkinson’s disease**	cKD (28 d)	PD (7)	Feasibility	Sustained ketosis with improved motor scores	[55]
	KD vs. low-fat diet (8 wk)	PD (47)	RCT	Both diets improved motor and non-motor symptoms; between-group advantage of the KD confined to non-motor symptoms	[24]
**Psychiatric disorders**	60:30:10 fat:protein:carbohydrate (4 mo)	Schizophrenia or bipolar disorder	Pilot	32% reduction in BPRS in schizophrenia; metabolic improvement	[26,56]
	KD	Bipolar disorder	Pilot	Acceptable and deliverable in outpatient care	[57]
**Heart failure**	3-hydroxybutyrate infusion	HFrEF (16)	Crossover	BHB 0.4→3.3 mmol/L; cardiac output +2.0 L/min; stroke volume +20 mL; effect from 0.7 mmol/L	[58]
**Type 2 diabetes**	Continuous-care nutritional ketosis (1 y)	T2D (262)	Non-randomized	HbA1c 7.6%→6.3%; weight −13.8 kg; insulin reduced or stopped in 94%	[59]
	Same program (5 y)	T2D (122 followed)	Non-randomized extension	Remission in 20%; HbA1c < 6.5% without medication or on metformin alone in 32.5%	[60]
	KD vs. Mediterranean diet (12 wk each)	Prediabetes/T2D (33)	Crossover RCT	No difference in HbA1c; LDL cholesterol +10% on KD	[61]
**MASLD/hepatic metabolism**	cKD (6 d)	Overweight adults (10)	Interventional, tracer	IHTG −31%; hepatic insulin resistance −58%; citrate synthase flux −38%; redox +167%	[62]
**Obesity**	KD	Adults with obesity	RCT	Improved mitochondrial respiration in circulating monocytes	[37]
**Type 2 diabetes (inflammation)**	Ketone monoester thrice daily (14 d)	T2D	Interventional	No change in plasma cytokines or leukocyte subsets	[63]
**Cancer**	KD (12 wk)	Locally advanced/metastatic breast cancer (80)	RCT	No difference in quality of life; serum lactate and ALP fell	[64]
	KD during radio-chemotherapy	Rectal cancer (18 vs. 23)	Controlled	Role, emotional, and social functioning maintained; approximately 50% found adherence difficult	[65]
	KD > 6 mo	Glioblastoma (6 adherent vs. 12)	Prospective	3-year survival in 4/6 vs. 8.3%	[66]

Abbreviations: AD, Alzheimer’s disease; ADAS-cog, Alzheimer’s Disease Assessment Scale–cognitive subscale; AE, adverse event; ALP, alkaline phosphatase; BHB, β-hydroxybutyrate; BPRS, Brief Psychiatric Rating Scale; cKD, classic ketogenic diet; CSF, cerebrospinal fluid; HbA1c, glycated hemoglobin; HFrEF, heart failure with reduced ejection fraction; IHTG, intrahepatic triglyceride; KD, ketogenic diet; KDT, ketogenic dietary therapy; LDL, low-density lipoprotein; LGIT, low glycemic index treatment; MAD, modified Atkins diet; MASLD, metabolic dysfunction-associated steatotic liver disease; MCI, mild cognitive impairment; MCT, medium-chain triglyceride; MELAS, mitochondrial encephalomyopathy, lactic acidosis, and stroke-like episodes; mtDNA, mitochondrial DNA; PD, Parkinson’s disease; RCT, randomized controlled trial; T2D, type 2 diabetes.

## Data Availability

The data supporting the findings of this study are available from the corresponding author upon reasonable request.

## Abbreviations

The following abbreviations are used in this manuscript:

AcAc	acetoacetate
AMPK	AMP-activated protein kinase
BDH1	D-β-hydroxybutyrate dehydrogenase 1
BHB	β-hydroxybutyrate
EC50	half-maximal effective concentration
FFAR3	free fatty acid receptor 3
GABA	γ-aminobutyric acid
HCAR2	hydroxycarboxylic acid receptor 2
HDAC	histone deacetylase
HMG-CoA	3-hydroxy-3-methylglutaryl-CoA
IL	interleukin
MCT1	monocarboxylate transporter 1
MCT2	monocarboxylate transporter 2
NAD	nicotinamide adenine dinucleotide
NLRP3	NLR family pyrin domain containing 3
SCOT	succinyl-CoA:3-oxoacid-CoA transferase
SIRT1	sirtuin 1
TCA	tricarboxylic acid
TFAM	mitochondrial transcription factor A
